# Evaluation of drying method and various level of chickpea powder on the quality characteristics of emulsified model sausages

**DOI:** 10.5713/ab.260060

**Published:** 2026-04-16

**Authors:** Min Jae Kim, Koo Bok Chin

**Affiliations:** 1 Department of Animal Science, Chonnam National University, Gwangju, Korea

**Keywords:** Chickpea, Concentrations, Emulsified Model Sausages, Freeze-drying, Oven-drying, Quality Characteristics

## Abstract

**Objective:**

This study evaluated the effects of chickpea powder (CPP) drying method (freeze-drying [FD] and oven-drying [OD]), and concentration (1.0% and 1.5%) on the quality characteristics of CPP-containing emulsified model sausages (EMSs), using a conventional emulsion-type meat system. CPP was assessed as a plant-based alternative to soy protein isolate (SPI), which is commonly used in meat formulations.

**Methods:**

CPPs were prepared by FD or OD at 60°C and incorporated into EMSs at 1.0% and 1.5%. A control without added protein (CTL) and an SPI-containing reference (REF) were included. Product pH, color, cooking loss (CL), expressible moisture (EM), proximate composition, texture profile parameters, protein solubility, surface hydrophobicity, and sulfhydryl group levels were analyzed. In addition, sodium dodecyl sulfate-polyacrylamide gel electrophoresis and scanning electron microscopy were performed.

**Results:**

CPP addition affected the pH, water-holding capacity, texture, and color of EMSs. Among the treatments, 1.5% oven-dried CPP showed the highest pH, lowest CL and EM, and the most improved textural properties. Redness decreased, whereas yellowness increased with CPP concentration, particularly in oven-dried CPP treatments. Fat content was unchanged, and protein content showed only minor differences. At 1.5%, CPP improved protein functionality by increasing protein solubility and reducing surface hydrophobicity and sulfhydryl groups, suggesting enhanced protein dispersion and cross-linking. Microstructural analysis confirmed a denser protein matrix with smaller pores and embedded starch granules. Overall, CPP-treated EMSs showed comparable or superior performance to SPI-treated reference EMSs.

**Conclusion:**

Both the drying method and concentration of CPP markedly affect EMS quality. Oven-dried CPP at 1.5% was especially effective in enhancing water retention and textural integrity, which was associated with improved protein functionality and microstructural stabilization, implying its usability as a functional plant-based protein ingredient in conventional emulsified meat products, expanding its applicability beyond reduced-fat systems.

## INTRODUCTION

Emulsified meat products such as sausages are widely consumed due to their palatability, convenience, and versatility [1]. Their physicochemical and textural qualities, most notably water and fat retention, emulsification, and gel formation, are greatly influenced by the functional properties of the proteins used during formulation [2]. In recent years, increasing consumer demand for healthier and more sustainable alternatives has prompted the food industry [3] to seek non-meat protein sources, such as soy, pea [4], faba bean [5], chickpea (CP) [6], that can replace or complement animal proteins in processed meat systems [7].

Currently, soy protein isolate (SPI) is widely used in meat products as a functional plant-based protein. However, there is growing interest in diversifying plant protein sources, as these novel functional ingredients possess unique compositional and structural properties. CP (*Cicer arietinum* L.) has attracted attention as a promising candidate due to its naturally high levels of globulin-type storage proteins (e.g., 7S and 11S) [8] and dietary fiber, as well as its value as a good source of carbohydrates [9]. Proteins in legumes, including CP, are primarily composed of globulin-type storage proteins, such as 7S and 11S globulins, which exhibit structural and functional similarities to soybean proteins. Among these, 7S globulin has been reported to interact with myofibrillar proteins (MPs), promoting interactions between plant and muscle proteins and contributing to the formation of protein networks [10,11]. The structural characteristics of globulins play a critical role in determining their functional properties, including emulsifying activity and water-holding capacity (WHC). Upon heating, globulin proteins undergo conformational changes that enhance intermolecular interactions, thereby inducing gel formation [11]. Because CP globulin exhibits molecular properties similar to those of soybean globulin [12], CP is also likely to show comparable functionality in meat systems. Wang et al [11] demonstrated that the interactions between 7S vicilin from CP protein isolate and MPs during thermal processing contribute to the formation of a stable protein gel network and improve WHC in meat batter systems. Furthermore, in emulsion systems, proteins play a crucial role in stabilizing oil droplets by forming interfacial films around dispersed lipids, thereby preventing undesirable phenomena such as aggregation, creaming, and phase separation [12].

CP protein exhibits favorable WHC and emulsifying ability, and it can potentially improve product texture [12], making it a suitable ingredient for meat analogs and hybrid meat products [13,14]. However, the functional properties of chickpea powder (CPP) can be affected by processing techniques, particularly the drying method [15]. Freeze-drying and oven-drying are two common techniques that influence the plant protein’s conformation, solubility, and interactions with meat proteins [16]. Oven-drying is a simple, safe, and cost-effective technique, while freeze-drying is particularly suitable for heat-sensitive foods, as it better preserves color, flavor, and nutritional value [16]. In addition, the concentration at which CPP is added may modulate its effects on the quality characteristics of meat products. According to previous studies, the functionality of CP varies depending on the level of addition. In our previous study, the incorporation of CP into the pork MP system improved gel properties, including viscosity, cooking yield, and gel strength, particularly at inclusion levels of 1.0% or higher, with 1.5% showing optimal performance [17]. Based on these findings, addition levels of 1.0% and 1.5% were selected in the present study to evaluate the applicability of CP in emulsified meat systems.

Although studies have evaluated the effect of incorporating CP-derived ingredients into meat matrices on product quality [6,18], investigations comparing meat matrices containing CPP at varying concentrations and processed via different drying methods remain limited. Furthermore, direct comparison with a commonly used reference protein, such as SPI, is required to assess the applicability of CPP as a functional alternative in emulsified meat systems. While previous studies have primarily focused on the application of CP-derived ingredients in protein gels or reduced-fat meat systems [17], the behavior of CPP in conventional emulsion-type sausages, where product quality is governed by the stability of a protein-fat-water matrix, remains insufficiently understood.

Therefore, the objective of this study was to evaluate the effects of drying methods (freeze-drying and oven-drying) and different CPP concentrations (1.0% and 1.5%) on the quality characteristics of emulsified model sausages (EMSs). An SPI treatment was included as a positive control to comparatively assess the functional properties of CPP and its potential as a plant-based protein substitute in meat products.

## MATERIALS AND METHODS

### Materials

Pork ham and back fat (1^st^ grade, Landrace×Yorkshire×Duroc crossbred) were obtained from a commercial meat supplier and used to prepare the EMSs. CPs were purchased from Hyundai Nongsan. After thorough washing, CPs were dried at 60°C for 24 h. For freeze-drying, samples were frozen at −70°C and dried at −50°C under vacuum conditions (<7 Pa) for 5 days. After drying, the moisture contents of oven-dried and freeze-dried CPPs were 5.43% and 5.52%, respectively. The dried samples were ground and passed through a 500 μm sieve to obtain powders with uniform particle size and were then stored at −70°C until use. SPI (SUPRO EX 33 IP, Solae) was used as a positive control (reference treatment), as it is widely used in the meat industry as a functional ingredient to improve water retention, emulsification, and binding properties. The inclusion of SPI enabled for the comparative evaluation of CPPs as a potential alternative functional protein in emulsified meat systems. Protein solubility of CPP and SPI powders was determined using the same salt extraction procedure and analytical method as applied to sausage samples (data not shown).

#### Preparation of emulsified model sausages

The formulations of EMSs are provided in Table 1. Freeze-dried or oven-dried CPPs were incorporated at concentrations of 1.0% and 1.5%, and the reference group (REF) EMSs contained 1.5% SPI, which was hydrated with distilled water at a ratio of 1:4 (w/v) prior to use. Thus, this study assessed four treatment groups, including EMSs containing freeze-dried CPP at 1.0% (Group F1) or 1.5% (Group F2) or oven-dried CPP at 1.0% (Group O1) or 1.5% (Group O2), comparing them to reference EMSs containing SPI at 1.5% (REF) and control sausages containing neither SPI nor CPP (CTL). Frozen pork and back fat were thawed at 4°C for 12 h before processing. The mixtures, composed of ground pork (60%), back fat (20%), ice water, non-meat ingredients (salt, STPP, sodium erythorbate, sodium nitrite), and the protein additives (SPI or CPP), were homogenized using a food mixer (SHMF-3000S; Hanil Electric) until forming a stable emulsion. The emulsified batters were then injected into 50 mL conical tubes and cooked in a water bath at 75°C for 30 min, at which point the internal temperature had reached 72°C. After cooking, the sausages were rapidly cooled in an ice bath and stored at 4°C until analysis.

#### Protein solubility

Protein solubility (%) of EMSs containing CPP produced using different drying methods and addition levels was determined using a slightly modified method based on Kim and Chin [19]. A 5 g sample of sausage batter was homogenized with 15 mL of salt solution (3% NaCl, 17.8 mM STPP, 1 mM NaN_3_, pH 6.0) for 30 s. The homogenate was stored at 4°C for 1 h to facilitate protein extraction, followed by centrifugation at 12,000×g for 30 min. The crude protein content (%) of the supernatant was determined using the Kjeldahl method. Protein solubility was calculated as follows:


(1) Protein solubility (%)=(Protein content of supernatant)×5/Protein content of sausage batter×100

#### Protein surface hydrophobicity

Protein surface hydrophobicity was determined according to the method of Chelh et al [20]. Cooked sausage samples were mixed with the salt solution, homogenized, and centrifuged, and the resulting supernatant was used for analysis. The protein concentration of the extracted solution was adjusted to 2 mg/mL. A bromophenol blue (BPB) solution (1 mg/mL bromophenol blue sodium salt) was prepared, and 40 μL of the BPB solution was added to 1 mL of the sample, followed by incubation for 10 min. A 20 mM sodium phosphate buffer (pH 6.0) was used as a control. After centrifugation at 8,000×g for 15 min, the absorbance of the supernatant was measured at 595 nm. BPB binding was calculated using the following equation:


(2) $$\text{BPB bound }(\mu \text{g})=40\;\mu \text{g}\times ({\text{A}}_{\text{control}}-{\text{A}}_{\text{sample}})/{\text{A}}_{\text{control}}$$

Where A represents the absorbance result.

#### Sulfhydryl group level

The sulfhydryl (SH) group content (μmol/g protein) was determined using 5,5′-dithiobis-(2-nitrobenzoic acid) (DTNB; Sigma-Aldrich) according to the method of Cui et al [21]. After cooking, proteins were extracted from sausage samples using a 2% SDS solution. The mixture was centrifuged, and the resulting supernatant was collected. The protein concentration was adjusted to 2 mg/mL prior to analysis.

For the determination of SH group content, 0.5 mL of the sample was mixed with 2.5 mL of Tris-glycine buffer containing 8 M urea and 20 μL of DTNB solution (4 mg/mL), followed by incubation for 30 min. After centrifugation at 12,000×g for 2 min, the absorbance of the supernatant was measured at 412 nm. The SH group content was calculated using the following equation:


(3) $$\text{SH group level }(\mu \text{mol}/\text{g protein})=73.53\times {\text{A}}_{\text{sample}}\times 6.04_{\text{dilution coefficient}}/2.00_{\text{protein concentration}}$$

Where A represents the absorbance result.

#### pH and color values

The pH of cooked EMS samples was measured using a solid-type pH meter (Model 340; Mettler-Toledo) by inserting the electrode directly into the sample. For each treatment, five measurements were conducted at different locations, and the average value was reported.

Color measurements were conducted on the cross-sectional surface of cooked EMS samples. Samples were sliced into 1.5 cm thick pieces, and color values were determined using a Minolta Color Reader (CR-10; Minolta). The CIE color-space L* (lightness), a* (redness), and b* (yellowness) parameter values were obtained from six random locations per sample, and the mean values were recorded.

#### Cooking loss

Cooking loss (CL, %) was determined by comparing the weight of EMS samples before and after cooking and cooling:


(4) CL (%)=([Sample weight before cooking-Sample weight after cooking]/Sample weight before cooking)×100

#### Expressible moisture

Expressible moisture (EM; %) was measured using a centrifugation method. Approximately 1.5 g of each cooked sample was weighed, wrapped in three layers of filter paper, and centrifuged at 1,660×g for 15 min using a centrifuge (VS-5500; Vision Science). The weight of the moisture released onto the filter paper was recorded, and EM was calculated as


(5) EM (%)=(Weight of released moisture [g]/ Sample weight [g])×100

#### Proximate analysis

Cooked samples were ground then moisture, crude fat, and crude protein contents were determined following the Association of Official Analytical Chemistry’s official methods [22]. Moisture content was measured by the dry oven method, crude fat content was analyzed using the Soxhlet extraction method, and crude protein content was determined using the Kjeldahl method.

#### Textural profile analysis

A texture profile analysis (TPA) was conducted using a universal testing machine (3344; Instron Corporation). Cooked sausage samples were cut into 1.25 cm-diameter, 1.3 cm-tall cylinders, with ten samples prepared per treatment, and the hardness (gf), springiness (mm), cohesiveness, gumminess, and chewiness of each sample was recorded. For each sample, the mean value of 10 measurements was used for analysis.

#### Sodium dodecyl sulfate-polyacrylamide gel electrophoresis

Sodium dodecyl sulfate-polyacrylamide gel electrophoresis (SDS-PAGE) was performed using a MINI-PROTEAN 3 Cell System (Bio-Rad Laboratories). Separating and stacking gels were prepared with 10% and 4% acrylamide, respectively. Sausage batter samples before cooking were dissolved in a salt solution, whereas cooked sausage samples were dissolved in a 5% SDS solution. The protein concentration of the supernatant from uncooked samples was determined using the Biuret method, while that of cooked samples was determined using a Pierce BCA protein assay kit (#23227; Thermo Fisher Scientific). Based on the measured protein concentrations, samples were mixed with 2× Laemmli sample buffer (#1610737; Bio-Rad Laboratories) to achieve a final loading of 15 μg per lane and then subjected to electrophoresis. Precision Plus Protein Standards (#161-0373; Bio-Rad Laboratories) were used as molecular weight markers.

#### Low-vacuum scanning electron microscopy

To investigate three-dimensional structural changes induced by cooking and the addition of non-meat proteins, low-vacuum scanning electron microscopy (LV-SEM) was performed on cooked EMSs. Samples were cut into cubes (approximately 3 mm^3^) and fixed in 2.5% glutaraldehyde at 4°C for 24 h. After fixation, the samples were washed with 0.1 M phosphate buffer (pH 7.0) for 10 min and then post-fixed in 1 % osmium tetroxide for 5 h. The samples were subsequently washed three times with the same phosphate buffer for 10 min each. Dehydration was performed by gradually increasing the ethanol concentration (50%, 60%, 70%, 80%, 90%, and 100%) at 10 min intervals, followed by immersion in acetone as a final pretreatment. After dehydration, the samples were dried for 24 h. The dried samples were sputter-coated with gold using an auto sputter coater (Model 108; Cressington Scientific Instruments), and the surface morphology was observed using an LV-SEM (JSM-6610V; JEOL).

#### Statistical analysis

All experiments were performed in triplicate as independent batches. Within each batch, multiple measurements (e.g., pH, color, and texture) were conducted as technical replicates, and the mean values were used for statistical analysis. All statistical analyses were performed using IBM SPSS Statistics software (ver. 29.0; IBM). One-way analyses of variance (ANOVAs) were conducted to assess the effects of treatments, and significant differences among means were identified using Duncan’s multiple range test. A p-value< 0.05 was accepted as indicating significance in all analyses.

## RESULTS AND DISCUSSION

### Protein solubility

Protein solubility is an important indicator of functional properties such as WHC, emulsifying ability, and gel formation [23]. The protein solubility results are presented in Table 2. In this study, protein solubility differed among treatments, with the F2 and O2 treatments (1.5% CPP) showing higher values than those of all other treatments (p<0.05). In contrast, no differences were observed among the CTL, REF, and treatments added with 1.0% CPP (F1 and O1) (p>0.05). These results indicate that CPP affects protein solubility when added above a certain threshold level.

Protein solubility in this study was determined using protein fractions extracted under salt solution conditions, representing a system in which MPs and CP proteins coexist. CP proteins are primarily composed of salt-soluble globulins (53%–60% of total CPP protein) [24], which exhibit high solubility under saline conditions. This characteristic is likely to be more pronounced at higher addition levels.

When evaluating the intrinsic solubility of the powders, CPP exhibited high solubility values (71.1% for freeze-dried CPP and 71.8% for oven-dried CPP), whereas SPI showed relatively low solubility (~17.2%). These results suggest that CP proteins possess high dispersibility, whereas the solubility of SPI may be limited by its structural characteristics and intermolecular interactions. Accordingly, the increased protein solubility observed in the sausage system with 1.5% CPP is likely attributable to the inherent properties of the CP proteins.

These findings are consistent with previous studies. Sha et al [25] reported that protein solubility in sausage decreased in the batter state upon SPI addition but showed no change after cooking, which was attributed to the relatively lower solubility of SPI compared to MP. Furthermore, 7S globulin in CP protein has been reported to interact with MP, contributing to protein network formation [11]. Such interactions are likely to occur more effectively when proteins are well dispersed. Therefore, the addition of CPP at 1.5% likely enhanced protein dispersibility and facilitated protein network formation during cooking, thereby improving functional properties.

Thus, the protein solubility of CPP-treated samples at 1.5% was comparable to or higher than that of the SPI-treated group, suggesting that CPP may serve as a potential alternative functional ingredient to SPI in emulsified meat systems. Meanwhile, protein solubility did not differ among treatments with different drying methods (p>0.05), suggesting that structural changes induced by drying had limited effects on protein solubility.

### Protein surface hydrophobicity

The results of protein surface hydrophobicity are presented in Table 2. Surface hydrophobicity differed among treatments, with CTL showing the highest value (p<0.05). In contrast, all CPP-added treatments exhibited lower values than CTL (p<0.05), and notably, F2 and O2 (1.5% CPP) showed levels comparable to REF (SPI) (p>0.05). These findings indicate that CPP addition reduced the exposure of hydrophobic regions in proteins.

In general, increased surface hydrophobicity enhances hydrophobic interactions among proteins, which can reduce dispersibility and consequently decrease protein solubility. Consistent with this relationship, CTL exhibited high hydrophobicity, whereas CPP-treated samples showed lower hydrophobicity accompanied by increased protein solubility, confirming an inverse association between these parameters. This trend agrees with previous reports indicating that protein solubility is governed by surface hydrophobicity and charge-related properties [25].

Because the analysis was performed on cooked EMS, these results reflect structural modifications induced during cooking. Similar trends have been reported previously; Kim and Chin [17] observed a decrease in surface hydrophobicity with increasing CPP levels in MP systems. This effect is likely associated with interactions between proteins and non-protein components, such as dietary fiber in CPP, which may limit the exposure of hydrophobic residues. Therefore, at the 1.0% CPP level, O1 showed lower hydrophobicity than F1 (p<0.05), whereas no differences were observed between drying methods at 1.5% CPP (p>0.05). This suggests that rearrangement and interactions during cooking.

### Sulfhydryl group

As shown in Table 2, sulfhydryl (-SH) group level varied among treatments (p<0.05). The CTL exhibited the highest value, whereas CPP-added treatments showed an overall decreasing trend, with the lowest values observed in F2 and O2 (1.5% CPP) (p<0.05). In addition, REF showed lower -SH group levels compared to CTL (p<0.05).

The reduction in -SH group level indicates the involvement of sulfhydryl groups in oxidation reactions, leading to the formation of disulfide bonds (S-S). Because measurements were conducted in cooked EMS, these changes reflect enhanced protein-protein interactions induced during cooking. In general, a decrease in -SH group level is associated with the formation of covalent bonds between proteins, which play a critical role in the development and stabilization of protein network structures.

This tendency is consistent with previous findings. Kim and Chin [17] reported that increasing CPP levels decreased -SH group level while promoting S-S bond formation, suggesting that CPP facilitates intermolecular cross-linking. Furthermore, 7S and 11S globulins, the major storage proteins in legumes, are known to contribute to gel network stability through disulfide bond formation [26]. In agreement with these observations, the reduction in -SH group level following CPP addition in the present study suggests enhanced formation of protein cross-links, accompanied by concurrent changes in protein dispersibility and functionality, as reflected in protein solubility and surface hydrophobicity. Meanwhile, at the 1.0% CPP level, -SH group level differed depending on the drying method (p<0.05), whereas no differences were observed at 1.5% CPP (p>0.05), indicating that the effect of drying was limited at higher inclusion levels. These modifications in protein cross-linking contribute to the formation of a stable gel network, which is closely associated with improvements in textural properties and WHC.

### pH and color values

The addition of CPP influenced the pH values of EMSs, with in the pH values of F2, O1, and O2 higher than those of the CTL (Table 3) (p<0.05). In contrast, previous studies have reported no changes in the pH of bologna sausages containing CP flour (as a model system) [27] or Merguez sausages containing CP protein concentrate [14], regardless of the levels of additive.

The plant-based protein additives affected sausage color. As shown in Table 3, the a* value was highest in the CTL, with gradual decreases observed following the addition of plant-based protein sources, whereas no differences were observed in L* values among treatments (p>0.05). Specifically, EMSs formulated with CPPs exhibited lower a* values than CTL EMSs (p<0.05), and the REF EMSs exhibited intermediate a* values that did not differ from those of CTL or some CPP-containing EMSs, indicating that SPI exerted a milder effect on redness than CPP. The b* value increased markedly with the addition of all CPPs (p<0.05). This increase in yellowness is likely attributable to the inherent yellow pigments in CPs, as yellowness appeared to increase with CPP concentration. In contrast, previous studies found that sausages formulated with faba bean protein isolate [5] and with pea or CP flour [28] showed no changes in color values, suggesting that the influence of plant protein ingredients on meat product color may vary depending on legume type and concentration.

### Cooking loss and expressible moisture

**Figure 1:**
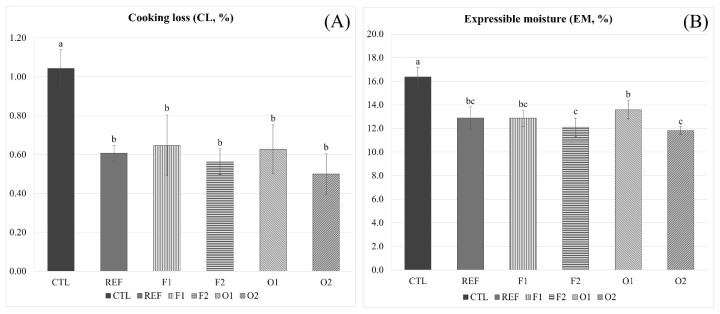
Cooking loss (CL, %) (A) and expressible moisture (EM, %) (B) of emulsified model sausages (EMS) added with different drying methods and various levels of chickpea powder (CPP). Treatment: CTL, EMS; REF, EMS added with 1.5% soy protein isolate; F1, EMS added with 1.0% freeze-dried CPP; F2, EMS added with 1.5% freeze-dried CPP; O1, EMS added with 1.0% oven-dried CPP; O2, EMS added with 1.5% oven-dried CPP. ^a–c^ Mean values with different superscripts are different among treatments, including different drying methods and CPP concentrations (p<0.05).

The CL and EM of EMSs incorporating SPI or CPP are presented in Figure 1. The CTL EMSs exhibited the highest CL (%) among all treatments (p<0.05), while no differences were observed among the SPI- and CPP-treated groups (p>0.05). This may be attributed to the increased water-binding capacity conferred by the added SPI and CPP, whereas excessive separation of fat and loss of moisture during heating could have contributed to CTL higher CL [28,29]. Furthermore, the incorporation of plant-based proteins may have contributed to the formation of a more cohesive protein matrix by filling gaps within the network structure, thereby limiting fluid migration and reducing CL [30]. These effects may also be associated with changes in protein functionality, including enhanced protein dispersion and network formation observed in the present study.

The lowest EM was observed in O2 (11.8%), followed by F2 (12.1%), both of which showed lower values than O1 (13.6%) (p<0.05). Additionally, the EM values of F1 (12.9%), F2 (12.1%), and O2 (11.8%) were not different from those of REF (12.9%), indicating that the CPPs exerted similar WHC to SPI, particularly at comparable concentration. This result suggests that CPP, particularly at 1.5% inclusion level, achieve WHC comparable to that of SPI, supporting its potential as an alternative functional ingredient in emulsified meat systems. These findings suggest that both the drying method and CPP concentration influence water retention in emulsion-type sausages. This improvement in WHC may be attributed to enhanced protein-protein interactions and water immobilization within the protein-fat-water matrix, as supported by changes in protein solubility, surface hydrophobicity, and sulfhydryl group content. In our previous study conducted using low-fat model sausages, reductions in CL and EM were primarily interpreted as improvements in water retention associated with fat replacement and protein gel reinforcement [17]. In contrast, the present results obtained from emulsion-type sausages indicate that changes in CL and EM reflect the stability of the protein-fat-water matrix during thermal processing, rather than a fat-reduction effect. This observation suggests that the functional behavior of CPP may vary depending on the meat product system. A similar trend was observed in a previous study, where the addition of CP flour at 2.5%–5.0% reduced EM compared to the control, regardless of CP type [27]. Likewise, the incorporation of pea protein isolates into chicken nuggets enhanced their WHC [31], and in another study, the incorporation of faba bean protein isolate into low-fat model sausages was shown to improve WHC and reduce EM by binding moisture within the meat matrix, ultimately increasing overall moisture content [5].

### Proximate composition

The proximate composition results for the EMSs, including moisture, fat, and protein content measures, are shown in Table 4. The addition of CPP into EMSs affected both moisture and protein content, while fat content remained unaffected (p>0.05). No differences between the moisture contents of CTL and O1 were observed, but F1, F2 and O2 exhibited lower moisture contents than CTL (p<0.05). Protein content did not differ among the CTL, REF, F1, F2, and O2 groups (p>0.05), but O1 exhibited a lower protein content than REF and F1 (p<0.05). These findings align with those of a previous study in which various levels of CP protein concentrate were incorporated into Merguez sausages with no effect on fat and protein content (p>0.05) [14]. Similarly, in a previous study on low-fat pork bologna (LFPB), the addition of CP flour at concentrations of 2.5% to 5.0% reduced moisture content in a concentration-dependent manner (p<0.05), while no differences were observed in protein content among treatments (p>0.05) [27]. In addition, fat content showed minimal variation, indicating that the addition of CP flour did not affect the fat retention. Thus, these results suggest that CP-derived ingredients have a limited impact on the overall proximate composition of meat products under the conditions of this study. This may be attributed to the comparable macronutrient profiles of CP proteins relative to meat proteins, particularly in terms of protein and fat contributions [32].

### Textural profile analysis

Texture plays a crucial role in determining the physical stability and sensory acceptability of meat products [33]. The texture profile properties of EMSs treated with CPPs are presented in Table 4. Hardness values were higher in all CPP-treated groups and the REF group than in the CTL group, with the highest values observed in the 1.5% CPP-treated groups (p<0.05). These results suggest that the incorporation of CPP improved gel network formation within the protein matrix, resulting in a firmer and more rigid structure, particularly at higher CPP concentrations (1.5%). This enhancement in textural properties may be associated with improved protein functionality, including increased protein solubility, reduced surface hydrophobicity, and enhanced disulfide bond formation, which contribute to the formation of a more stable and cohesive protein network. This is consistent with the findings of Petridis et al [34], who reported that the gel properties of frankfurters vary with protein content and that higher protein levels promote the formation of a firmer and more cohesive gel network. Springiness was higher in REF and both 1.5% CPP-treated groups than in the CTL group (p<0.05), while the 1.0% CPP-treated groups exhibited springiness similar to that of CTL (p>0.05). Additionally, gumminess, chewiness, and cohesiveness were higher in all SPI- and CPP-treated groups than in the CTL group (p<0.05), with these textural characteristics increasing as the CPP concentration increased. These findings suggest that the incorporation of CPPs improved the overall textural integrity of the EMSs by promoting stronger protein–protein interactions and forming more stable gel networks, which are critical for the structural quality of emulsified meat products. The observed changes in sulfhydryl groups, protein solubility, and microstructure indicate that CP proteins participate in intermolecular interactions with MPs during heating, promoting network formation and improving textural properties. Similarly, a previous study reported that the addition of CP protein isolate (CPI) increased in hardness and chewiness [35], which was attributed to the formation of a compact and homogeneous gel matrix between CPI and meat proteins during heating, thereby enhancing the texture of meat products [11,35]. Similar increases in textural attributes following the incorporation of CP-derived ingredients have been reported. For example, sausages formulated with cooked CP paste exhibited higher hardness, cohesiveness, and chewiness than those with raw CP paste [6], regardless of the potential for reductions in protein gelation functionality during pre-cooking [36]. In contrast to low-fat meat systems, where textural changes are often attributed to fat replacement and reinforcement of protein gels, the improvements observed in the present emulsion-type sausages reflect enhanced structural integrity of the protein-fat-water matrix, which contributes to the stability of conventional emulsified products. Notably, several quality attributes of CPP-treated samples, particularly at a 1.5% inclusion level, were comparable to those of the SPI-treated group, suggesting that CPP may serve as a potential alternative functional ingredient in emulsified meat systems under the conditions of this study.

### Sodium dodecyl sulfate-polyacrylamide gel electrophoresis

**Figure 2:**
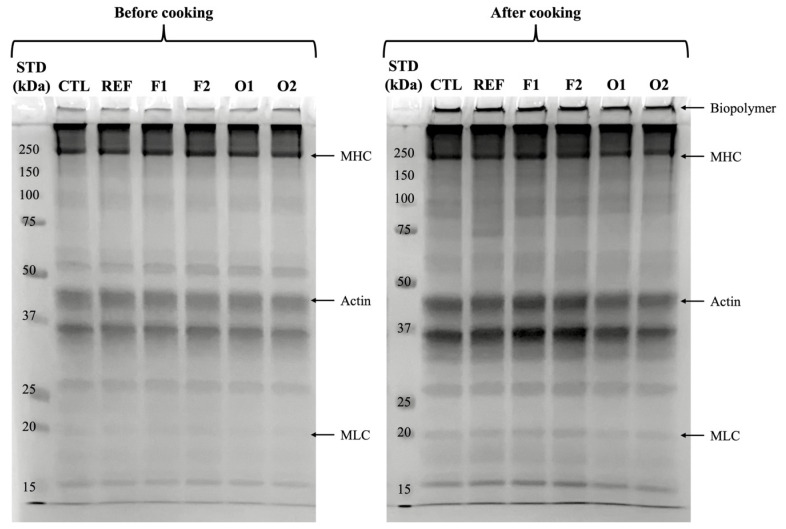
SDS-PAGE patterns of emulsified model sausages (EMS) added with different drying methods and various levels of chickpea powder (CPP). STD: protein standard marker; Treatment: CTL, EMS; REF, EMS added with 1.5% soy protein isolate; F1, EMS added with 1.0% freeze-dried CPP; F2, EMS added with 1.5% freeze-dried CPP; O1, EMS added with 1.0% oven-dried CPP; O2, EMS added with 1.5% oven-dried CPP. MHC, myosin heavy chain; MLC, myosin light chain; SDS-PAGE, sodium dodecyl sulfate-polyacrylamide gel electrophoresis.

Figure 2 illustrates the protein patterns of EMSs before and after cooking. Prior to cooking, protein bands corresponding to myosin heavy chain (MHC), actin, and myosin light chain (MLC) were consistently detected across all treatments, regardless of CPP levels or drying method, indicating that CPP incorporation did not alter the initial protein composition. Following cooking, MHC band intensity decreased, accompanied by the accumulation of high-molecular-weight aggregates at the top of the stacking gel. These changes reflect heat-induced denaturation of MPs, leading to protein unfolding and subsequent aggregation through enhanced protein-protein interactions. Such behavior is consistent with previous reports describing the formation of polymerized aggregates and three-dimensional gel networks during thermal processing [37]. In particular, myosin and actin are known to form insoluble aggregates that contribute to the development of gel structure.

Despite these structural transitions, the electrophoretic profiles remained comparable among treatments, suggesting that CPP did not substantially modify protein band distribution. Instead, CPP likely functioned as a supplementary component that facilitates intermolecular interactions during network formation. This interpretation is supported by the observed decrease in sulfhydryl group content and changes in protein functionality, which are indicative of increased protein cross-linking. Although previous studies have reported increased high-molecular-weight aggregates with higher CPP levels [17], no distinct differences were observed in the present study. This may be attributed to the complex emulsion matrix, where the coexistence of fat and water phases can obscure subtle differences in protein interactions in SDS-PAGE analysis.

### Low-vacuum scanning electron microscopy

**Figure 3:**
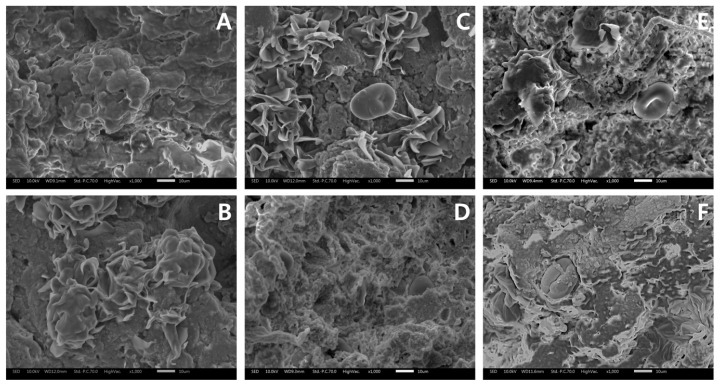
Scanning electron micrographs (×1,000 magnification) of emulsified model sausages (EMS) added with different drying methods and various levels of chickpea powder (CPP). Treatment: (A) CTL, EMS; (B) REF, EMS added with 1.5% soy protein isolate; (C) F1, EMS added with 1.0% freeze-dried CPP; (D) F2, EMS added with 1.5% freeze-dried CPP; (E) O1, EMS added with 1.0% oven-dried CPP; (F) O2, EMS added with 1.5% oven-dried CPP.

Figure 3 presents the surface microstructure of EMSs. The CTL sample (A) exhibited a relatively coarse and heterogeneous structure with large pores. These pores are likely formed by the migration of moisture and entrapped air during cooking and may serve as channels for water release, thereby contributing to reduced WHC and increased CL [33].

In contrast, the REF sample (B) containing SPI and the CPP-treated samples (C–F) showed more compact and homogeneous structures, accompanied by a reduction in pore size. Notably, samples with 1.5% CPP (F2 and O2) exhibited denser microstructures than those with 1.0% CPP (F1 and O1). This structural refinement may be attributed to the filling of pores within the protein matrix and enhanced protein-protein interactions induced by CPP addition. These observations are consistent with previous studies reporting that plant protein incorporation reduces pore size and promotes structural densification [18,38].

Additionally, smooth spherical or elliptical particles were observed in CPP-treated samples. These structures are likely derived from starch components present in CPP and appear to remain embedded within the protein matrix after cooking. Starch granules are typically spherical or elliptical in shape with micrometer-scale dimensions [39], supporting this interpretation. The presence of these particles may contribute to filling pores within the matrix, thereby enhancing structural compactness. This effect is also associated with improved WHC, as evidenced by the reductions in CL and EM observed in this study.

## CONCLUSION

The drying method and inclusion level of CPP influenced the quality characteristics of EMSs. In particular, the incorporation of 1.5% CPP improved WHC as evidence by lower CL and EM, and textural properties. These improvements were associated with enhanced protein functionality, including increased protein solubility, reduced surface hydrophobicity, and decreased sulfhydryl group level, indicating strengthened protein network formation. In addition, microstructural analysis revealed more compact and homogeneous structure in CPP treatments. Overall, CPP treatments exhibited quality characteristics comparable to those of SPI treatment, suggesting that CPP may serve as a functional plant-based protein ingredient in emulsified meat products under the conditions of this study.

## Figures and Tables

**Table 1:** Formulation of emulsified model sausages (EMSs) with different drying methods and various levels of chickpea powder (CPP)

Ingredients (%)	Treatment^1)^					

	CTL	REF	F1	F2	O1	O2
1. Meat	60.0	60.0	60.0	60.0	60.0	60.0
2. Fat	20.0	20.0	20.0	20.0	20.0	20.0
3. Water	18.13	18.13	18.13	18.13	18.13	18.13
^1)^ Ice water	18.13	12.13	18.13	18.13	18.13	18.13
2) Hydrate water	0.00	6.00	0.00	0.00	0.00	0.00
4. NMI	1.87	3.37	2.87	3.37	2.87	3.37
^1)^ Salt	1.50	1.50	1.50	1.50	1.50	1.50
2) STPP	0.30	0.30	0.30	0.30	0.30	0.30
3) Sodium erythorbate	0.05	0.05	0.05	0.05	0.05	0.05
4) Sodium nitrite	0.015	0.015	0.015	0.015	0.015	0.015
5) Soy protein isolate	0.00	1.50	0.00	0.00	0.00	0.00
6) CPP	0.00	0.00	1.00	1.50	1.00	1.50
(^1)^ Freeze-drying	0.00	0.00	1.00	1.50	0.00	0.00
(2) Oven drying	0.00	0.00	0.00	0.00	1.00	1.50
Total	100.0	101.5	101.0	101.5	101.0	101.5

1) Treatment: CTL, EMS; REF, EMS added with 1.5% soy protein isolate; F1, EMS added with 1.0% freeze-dried CPP; F2, EMS added with 1.5% freeze-dried CPP; O1, EMS added with 1.0% oven-dried CPP; O2, EMS added with 1.5% oven-dried CPP.

NMI, non-meat ingredients; STPP, sodium tripolyphosphate.

**Table 2:** Protein solubility, protein surface hydrophobicity, and SH group level of emulsified model sausages (EMS) added with different drying methods and various levels of chickpea powder (CPP)

	Protein solubility (%)	Hydrophobicity (Bound BPB, μg)	-SH group level (μmol/g protein)
CTL	46.5±2.12^b^	6.61±0.34^a^	129±1.70^a^
REF	46.5±0.52^b^	3.73±0.15^d^	110±2.50^d^
F1	46.1±0.58^b^	5.76±0.22^b^	120±1.28^b^
F2	49.7±0.68^a^	3.81±0.17^d^	107±2.12^e^
O1	47.5±1.76^b^	5.16±0.09^c^	116±0.44^c^
O2	50.8±0.21^a^	3.71±0.23^d^	107±1.89^e^

Treatment: CTL, EMS; REF, EMS added with 1.5% soy protein isolate; F1, EMS added with 1.0% freeze-dried CPP; F2, EMS added with 1.5% freeze-dried CPP; O1, EMS added with 1.0% oven-dried CPP; O2, EMS added with 1.5% oven-dried CPP.

a–e Mean values with different superscripts are different among treatments, including different drying methods and CPP concentrations (p<0.05).

**Table 3:** pH and color values of emulsified model sausages (EMS) added with different drying methods and various levels of chickpea powder (CPP)

CTL	Treatment^1)^					

	REF	F1	F2	O1	O2	
pH	6.11±0.01^c^	6.15±0.01^b^	6.11±0.02^c^	6.14±0.01^b^	6.14±0.01^b^	6.17±0.00^a^
CIE L* (lightness)	73.9±1.42^a^	73.9±2.26^a^	74.1±1.26^a^	73.3±0.82^a^	74.5±1.69^a^	74.5±1.57^a^
CIE a* (redness)	8.62±0.33^a^	8.26±0.56^ab^	7.47±0.44^bc^	7.51±0.63^bc^	7.53±0.68^bc^	6.86±0.41^c^
CIE b* (yellowness)	7.25±0.14^c^	8.13±0.23^b^	8.26±0.14^b^	8.48±0.23^ab^	8.16±0.31^b^	8.73±0.10^a^

1) Treatment: CTL, EMS; REF, EMS added with 1.5% soy protein isolate; F1, EMS added with 1.0% freeze-dried CPP; F2, EMS added with 1.5% freeze-dried CPP; O1, EMS added with 1.0% oven-dried CPP; O2, EMS added with 1.5% oven-dried CPP.

a–c Mean values with different superscripts are different among treatments, including different drying methods and CPP concentrations (p<0.05).

**Table 4:** Proximate composition and texture profile analysis of emulsified model sausages (EMS) added with different drying methods and various levels of chickpea powder (CPP)

CTL	Treatment^1)^					

	REF	F1	F2	O1	O2	
Moisture (%)	66.4±0.35^a^	66.0±0.55^ab^	65.4±0.44^bc^	65.0±0.24^c^	66.3±0.27^a^	65.4±0.18^bc^
Fat (%)	17.7±0.24^a^	17.4±0.38^a^	17.9±0.75^a^	17.6±0.76^a^	17.6±0.67^a^	17.8±0.72^a^
Protein (%)	13.0±0.03^ab^	13.3±0.21^a^	13.2±0.25^a^	13.1±0.35^ab^	12.5±0.20^b^	13.0±0.45^ab^
Hardness (gf)	3,873±94.5^d^	5,389±56.2^b^	5,089±103^c^	5,572±49.7^a^	5,066±126^c^	5,644±14.9^a^
Springiness (mm)	5.02±0.34^c^	5.35±0.14^ab^	5.16±0.01^bc^	5.34±0.07^ab^	5.18±0.02^bc^	5.55±0.03^a^
Gumminess	32.8±1.08^d^	47.2±1.34^b^	44.4±1.11^c^	51.3±0.93^a^	45.4±1.63^bc^	52.7±0.88^a^
Chewiness	165±15.2^e^	252±5.60^c^	230±6.35^d^	274±8.79^b^	235±7.23^d^	293±3.61^a^
Cohesiveness	0.85±0.01^c^	0.88±0.02^b^	0.88±0.00^b^	0.92±0.01^a^	0.90±0.01^b^	0.93±0.01^a^

1) Treatment: CTL, EMS; REF, EMS added with 1.5% soy protein isolate; F1, EMS added with 1.0% freeze-dried CPP; F2, EMS added with 1.5% freeze-dried CPP; O1, EMS added with 1.0% oven-dried CPP; O2, EMS added with 1.5% oven-dried CPP.

a–e Mean values with different superscripts are different among treatments, including different drying methods and CPP concentrations (p<0.05).

## Data Availability

Upon reasonable request, the datasets of this study can be available from the corresponding author.
